# Emerging CCR5-Based Therapeutic Potential of JAK/STAT Inhibitors and CCR5Δ32 Hematopoietic Stem Cell Transplantation

**DOI:** 10.3390/v18090933

**Published:** 2026-08-27

**Authors:** Khadija Khalid, Uzair Iqbal, Mohamed Shaltout, Yunus Yukselten, Farooq Ahmad, Abdur Rehman Khalid, Richard E. Sutton

**Affiliations:** Section of Infectious Diseases, Department of Medicine, Yale School of Medicine, New Haven, CT 06520, USA; khadija.khalid@yale.edu (K.K.); uzair.iqbal@yale.edu (U.I.); mohamed.shaltout@yale.edu (M.S.); yunus.yukselten@yale.edu (Y.Y.); farooq.ahmad@yale.edu (F.A.); abdurrehman.khalid@yale.edu (A.R.K.)

**Keywords:** CCR5, CCR5Δ32, HIV cure, hematopoietic stem cell transplantation (HSCT), JAK/STAT signaling, JAK inhibitors, immune modulation

## Abstract

C-C chemokine receptor type 5 (CCR5) is the primary co-receptor mediating the entry of R5-tropic human immunodeficiency virus type 1 (HIV-1) into CD4+ T cells and macrophages, making it one of the most promising therapeutic targets in HIV cure research. The naturally occurring CCR5Δ32 mutation which abolishes surface CCR5 expression in homozygous individuals has provided strong clinical evidence for CCR5-directed strategies after multiple cases of sustained HIV remission following allogeneic hematopoietic stem cell transplantation (HSCT) from CCR5Δ32 donors. This review summarizes the current understanding of the evolutionary origin and global distribution of the CCR5Δ32 allele, as well as the clinical evidence from landmark transplantation cases. We also discuss recent findings demonstrating that durable HIV remission may be achieved following transplantation from heterozygous CCR5 wild-type/Δ32 donors, suggesting that factors such as donor chimerism, conditioning regimens and graft-versus-reservoir effects contribute substantially to reservoir clearance alongside CCR5 disruption. In addition, we examine emerging evidence that pharmacological inhibition of the Janus kinase/signal transducer and activator of transcription (JAK/STAT) pathway can reversibly suppress CCR5 expression, reduce HIV replication, limit reservoir establishment, and attenuate immune activation. Collectively, these advances highlight the evolving landscape of CCR5-targeted therapies and support the development of safer and more broadly applicable approaches toward durable HIV remission and, ultimately, an HIV cure.

## 1. Introduction

Human immunodeficiency virus type 1 (HIV-1) remains a major global health burden. Although combination antiretroviral therapy (ART) reliably suppresses viremia, it does not eradicate the long-lived latent reservoir and lifelong treatment is required to prevent viral rebound [1,2]. This limitation has driven sustained interest in curative strategies and among the many host factors implicated in HIV-1 pathogenesis, the C-C chemokine receptor type 5 (CCR5) has emerged as the single most validated therapeutic target [3].

CCR5 is the principal co-receptor mediating entry of R5-tropic HIV-1 into CD4+ T cells and macrophages, functioning together with CD4 during the earliest stages of transmission and acute infection [4,5]. Its central role is underscored by a naturally occurring 32-base-pair deletion, CCR5∆32, which introduces a frameshift that abolishes surface expression of the receptor. Homozygous carriers display near-complete resistance to R5-tropic HIV-1 infection despite exposure, whereas heterozygotes exhibit reduced receptor density and slower disease progression [5,6]. The allele shows a striking geographic gradient, reaching approximately 10% frequency in populations of European descent while remaining nearly absent in sub-Saharan African, East Asian and Native American populations, a distribution attributed to relatively recent positive selection [6]. Because homozygous CCR5∆32 confers a heritable, loss-of-function block to viral entry without apparent major clinical detriment in most carriers, it has become the conceptual foundation for CCR5-directed cure research [3,5].

The clinical proof of concept came from allogeneic hematopoietic stem cell transplantation (allo-HSCT). Beginning with a Berlin patient, at least five individuals have achieved durable, ART-free HIV-1 remission after transplantation from CCR5∆32/∆32 donors for hematological malignancies, including London, Düsseldorf, New York and City of Hope patients, with additional cases reported subsequently [6,7,8]. In-depth virological and immunological characterization of these individuals have repeatedly demonstrated an absence of replication-competent virus, waning HIV-specific immune responses and no rebound after analytical treatment interruption, consistent with a functional cure [7,9]. These cases established that reconstitution of the immune system with CCR5-deficient donor cells can render the graft refractory to R5-tropic HIV-1 and enable profound reservoir depletion [10].

However, the mechanistic outlook has grown more nuanced. Observational data from the IciStem cohort showed that substantial reservoir reduction also occurs after transplantation with CCR5 wild-type donor cells, implicating transplantation-associated factors such as full donor chimerism, conditioning intensity and a graft-versus-reservoir effect in reservoir clearance independent of CCR5 status [11,12]. This shift was reinforced by a landmark 2026 report of sustained HIV-1 remission for more than six years after allo-HSCT from a heterozygous CCR5 wild-type/∆32 donor, in a recipient whose reconstituted immune system expressed functionally active CCR5 [13]. Robust antibody-dependent cellular cytotoxicity at the time of transplantation was proposed as a key contributor to reservoir clearance, demonstrating that complete CCR5 ablation is not strictly essential for durable remission [13]. Collectively, these findings reframe CCR5∆32 as one important component of a multifactorial curative process rather than its sole determinant.

Despite these successes, allo-HSCT is neither scalable nor safe enough to apply to the tens of millions of people living with HIV, given transplant-related morbidity, the rarity of HLA-matched CCR5∆32/∆32 donors and residual tropism-related risks from archived CXCR4-using virus [3,14]. These constraints have motivated the search for reversible, pharmacologically accessible means of downregulating CCR5 that could recapitulate elements of the transplantation experience without its toxicity. In this context, the Janus kinase/signal transducer and activator of transcription (JAK/STAT) pathway has attracted increasing attention. The CCR5 promoter contains functional STAT-binding sites and cytokine-driven JAK/STAT signaling positively regulates CCR5 transcription in primary CD4+ T cells [2]. Pharmacological JAK/STAT inhibition with agents such as ruxolitinib and tofacitinib reversibly suppresses CCR5 expression including in cells from viremic HIV-positive patients and CRISPR-mediated knockout of JAK2, STAT3, STAT5A and STAT5B confirms direct transcriptional control of CCR5 by this axis [2]. Beyond receptor modulation, JAK inhibitors exert sub-micromolar inhibition of HIV-1 replication, limit reservoir seeding, block cytokine-induced reactivation and attenuate the immune activation that sustains viral persistence [15,16,17].

This review integrates the evolutionary and population genetics of CCR5∆32, the clinical evidence from landmark transplantation cures and the emerging recognition of CCR5-independent mechanisms of reservoir clearance with a focus on the therapeutic potential of JAK/STAT-mediated CCR5 modulation. By situating reversible pharmacological CCR5 downregulation alongside the lessons of CCR5∆32 transplantation, we aim to outline safer, more broadly applicable strategies for advancing toward durable HIV-1 remission and ultimately a cure.

## 2. Population Distribution and Evolutionary Origins of CCR5Δ32

The CCR5Δ32 allele exhibits a striking geographic gradient. The highest heterozygous frequencies (~10–16%) are observed in European-descent populations, with the peak homozygous frequency (2.3%) recorded in the Faroe Islands [18]. The allele is nearly absent in sub-Saharan African, East Asian and Native American populations [19]. In Latin America, frequencies range from 1.29% in Colombia to 5.46% in Argentina, reflecting post-Columbian European combination [20,21]. Recent ancient genomic analyses traced the origin of the CCR5Δ32 deletion to at least 6700 years before the present in the Western Eurasian Steppe, with strong evidence for positive selection between 8000 and 2000 years ago [21]. Earlier hypotheses attributed this selective pressure to resistance against Yersinia pestis, or the plague bacillus, during the 14th-century Black Death, although the revised timeline suggests earlier selective forces may have been operational [6,21]. The rarity of homozygous donors (~0.27–1% of European-descent populations) poses a significant logistical challenge for identifying HLA-matched CCR5Δ32/Δ32 donors for transplantation [20].

## 3. Landmark Cases of HIV Cure Through CCR5Δ32 Stem Cell Transplantation

The Berlin patient reported in 2009 was the first individual considered cured of HIV-1 after receiving two allogeneic hematopoietic stem cell transplants (allo-HSCTs) from a CCR5Δ32/Δ32 donor for acute myeloid leukemia (AML), with myeloablative conditioning that included total body irradiation [20,22]. He maintained undetectable viral loads without antiretroviral therapy (ART) until his death from recurrent AML 13 years later [20]. The London patient, Adam Castillo who was born in Venezuela, demonstrated that a less aggressive approach, a single allo-HSCT with reduced-intensity conditioning and no irradiation from a CCR5Δ32/Δ32 donor for Hodgkin’s lymphoma could also achieve sustained HIV-1 remission [22]. The Düsseldorf patient provided the most extensively characterized case, with more than 9 years of monitoring after CCR5Δ32/Δ32 HSCT for AML showing no replication-competent virus despite sporadic traces of HIV-1 DNA, declining HIV-specific immune responses and four years of sustained remission after analytical treatment interruption [7]. Additional cases include the New York patient, who received a CCR5Δ32/Δ32 haplo-cord transplant (cord blood cells combined with haploidentical adult stem cells) for AML, the first remission in a woman of mixed-race ancestry with 100% CCR5Δ32/Δ32 cord blood chimerism by week 14 and sustained undetectable HIV-1 load, in the absence of antiretroviral therapy [8]. The City of Hope patient, treated at age 63, demonstrated that older patients receiving reduced-intensity conditioning could also achieve HIV cure [22]. Most recently, a 63-year-old man has been in remission from HIV-1 five years after HSCT with a CCR5Δ32/Δ32 sibling donor for myelodysplastic syndrome, with full donor chimerism in gut tissue (the primary viral reservoir) and absent replication-competent virus [9]. All these cases are summarized in the Table 1 as follows:

## 4. Mechanisms of HIV-1 Reservoir Clearance Beyond CCR5Δ32

**Figure 1:**
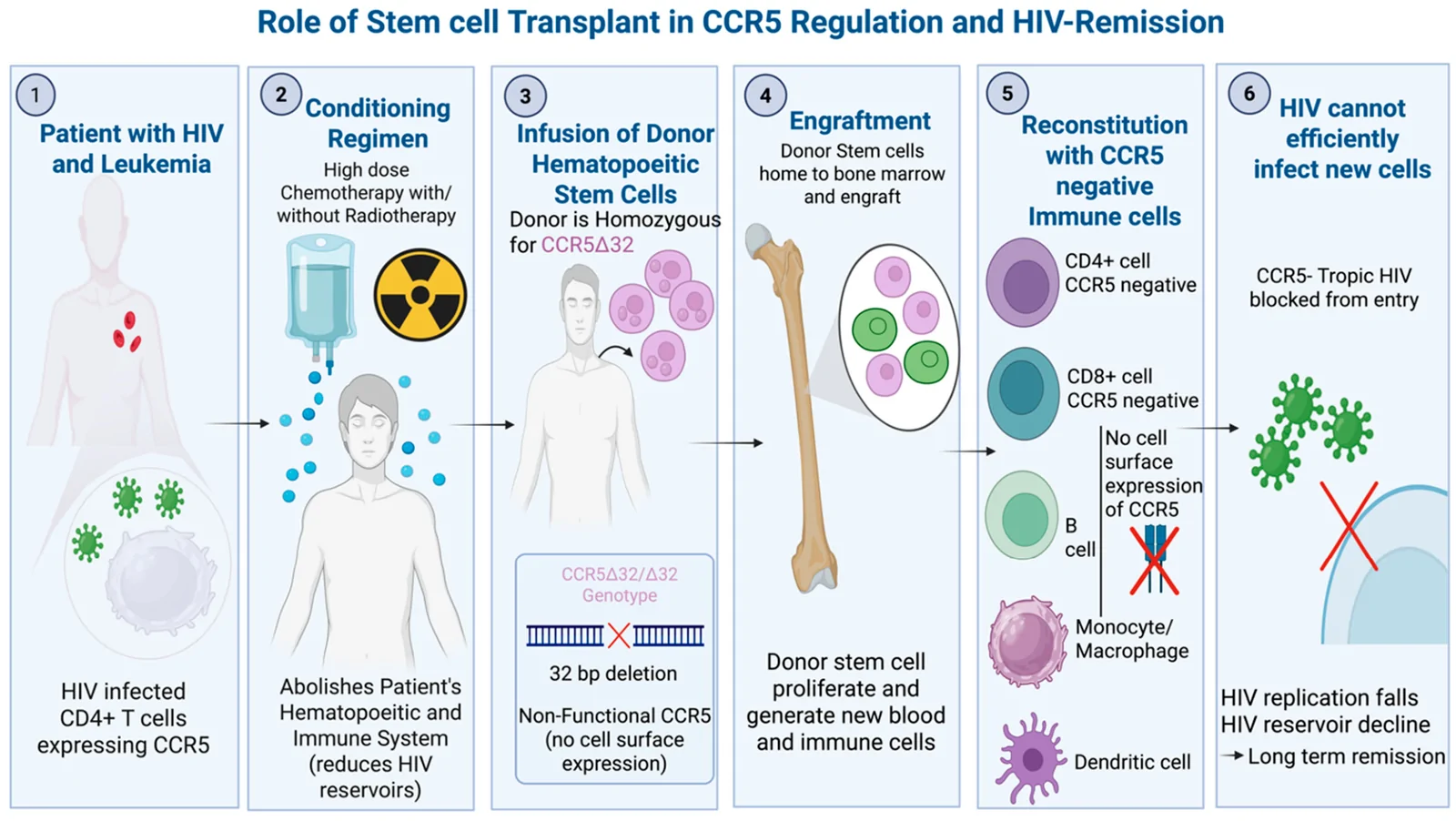
HIV-1 remission following CCR5Δ32 hematopoietic stem cell transplantation.

While CCR5Δ32/Δ32 donor cells prevent graft infection with R5-tropic HIV, emerging evidence indicates that CCR5-independent mechanisms contribute critically to HIV cure. The IciStem consortium, the largest observational cohort of 30 HIV-positive transplant recipients demonstrated that HIV-1 reservoir reduction after allo-HSCT was comparable regardless of whether donors were CCR5Δ32/Δ32 or CCR5 wild-type. This suggests that the transplant procedure itself drives reservoir depletion rather than donor CCR5 status alone [11]. Complete donor chimerism appears to be an important factor associated with Durable HIV remission and reservoir reduction, although it is not clearly established as an absolute requirement for HIV cure [23]. A paradigm-shifting case reported in 2025/2026 demonstrated sustained HIV-1 remission for more than 6 years after allo-HSCT from a heterozygous CCR5 wild-type/Δ32 donor, despite intact HIV-1 proviral DNA being detectable prior to transplantation [13]. Because the reconstituted immune system expressed functionally active CCR5, this case offered a unique window into CCR5-independent clearance mechanisms: the patient exhibited an unusually high proportion of adaptive NKG2A+NKG2C−CD57+ natural killer (NK) cells and markedly elevated antibody-dependent cellular cytotoxicity (ADCC) activity around the time of transplantation, with degranulation responses (79.3% pre-transplant, 74.8% post-transplant) exceeding those typically observed in elite controllers. Both HIV-specific antibody titers and ADCC activity subsequently declined over years of follow-up in parallel with loss of detectable HIV-specific T-cell responses, consistent with waning antigen exposure rather than ongoing viral replication. These findings implicate enhanced innate, antibody-dependent NK cell immunity as an important CCR5-independent contributor to HIV-1 reservoir clearance after transplantation [13] (Figure 1).

## 5. Limitations of CCR5-Based Strategies

One of the challenges faced even after CCR5Δ32 HPSCT is that HIV still infects by using CXCR4-Tropic. HIV-1 entry requires gp120 engagement of CD4 followed by binding to a chemokine co-receptor, either CCR5 (R5 virus), CXCR4 (X4 virus) or both (dual-tropic). A patient may also harbor a mixed population of variants with differing tropism (collectively dual/mixed, D/M) [24]. The V3 loop of gp120 is the principal determinant of co-receptor specificity [21]. Because every CCR5-directed intervention like pharmacologic antagonism, CCR5∆32 transplantation or gene editing acts exclusively on the R5 entry pathway, none of these strategies constrains virus capable of using CXCR4. This represents a fundamental biological ceiling on all CCR5-based approaches [25].

## 6. Natural History of R5-to-X4 Tropism Switching

R5 variants are preferentially transmitted and predominate in early infection, whereas X4 and D/M variants emerge later and are strongly associated with accelerated CD4+ T-cell decline and disease progression [24,25,26]. Up to 19% of individuals with acute or recent infection can already harbor CXCR4-tropic virus and longitudinal cohorts show tropism switching in roughly 18% of recently infected individuals, with a mean evolution time of approximately 27 months and a low baseline geno2pheno false-positive ratio as the strongest predictor [27]. Critically, the prevalence of X4- or D/M-tropic variants rises to more than 50% in treated patients with CD4 counts <100 cells/mm^3^ and antiretroviral-experienced patients with extensive resistance or persistent viremia are enriched for such variants [28]. Immune activation appears to precede and independently predict X4 emergence, suggesting the inflammatory milieu of advanced infection selects for CXCR4 usage [25,29]. Because HIV cure candidates are frequently long-standing, treatment-experienced individuals with prior immunosuppression, the pre-transplant probability of an archived X4 reservoir is not negligible.

## 7. Clinical Evidence: X4 Rebound After CCR5∆32/∆32 Transplantation

**Figure 2:**
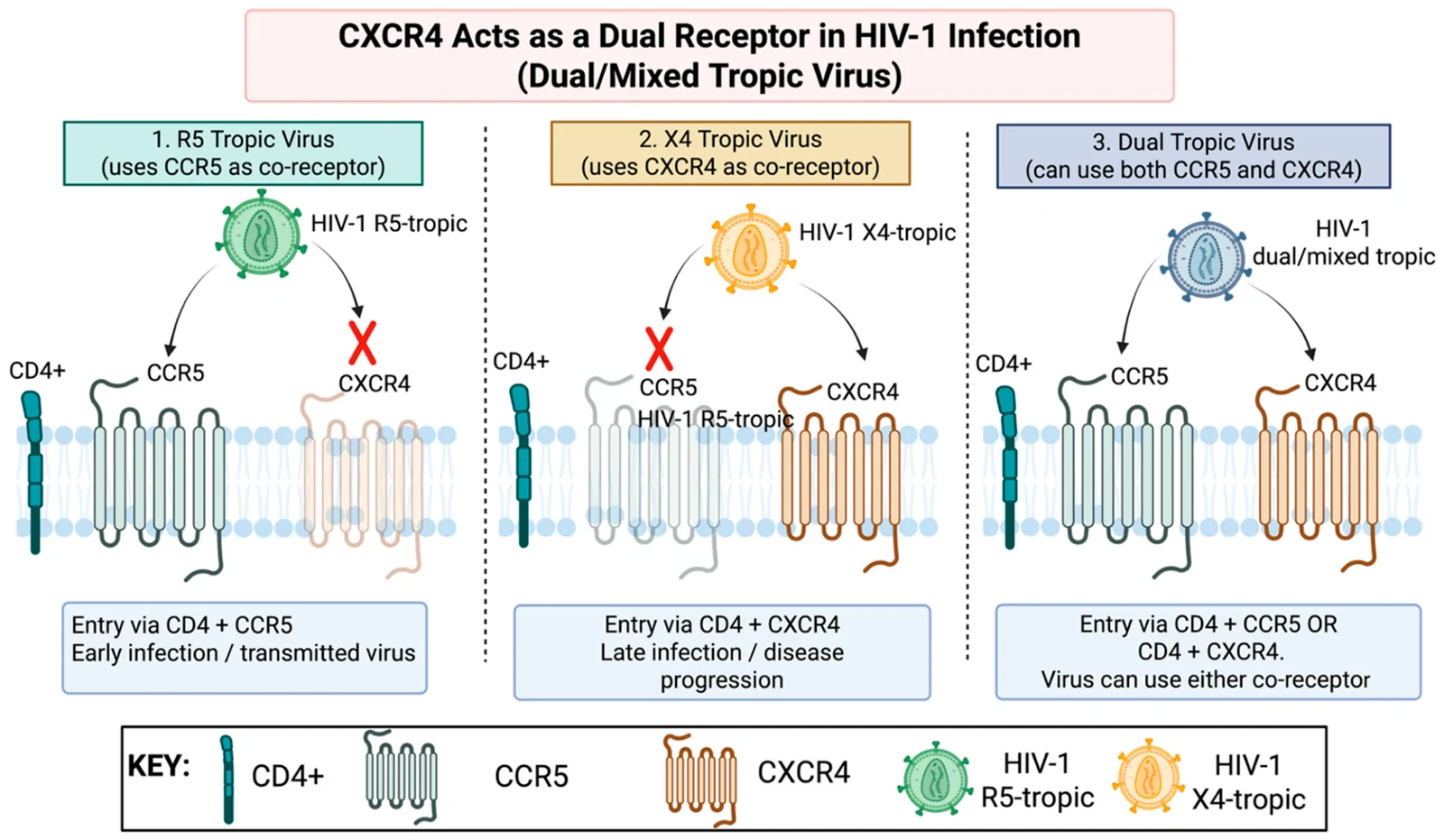
CXCR4 as a dual receptor for HIV-1 infection.

The most direct demonstration that CCR5 ablation does not protect against all HIV variants comes from a patient who underwent allogeneic transplantation with CCR5∆32/∆32 homozygous stem cells and experienced rapid viral rebound driven by a highly replicative CXCR4-tropic variant that was detectable at low levels before transplantation [30]. The reconstituted CCR5-negative immune compartment, while resistant to R5 virus, remained fully susceptible to the pre-existing X4 population, which expanded under the selective pressure of CCR5 absence [30,31] (Figure 2). This case contrasts instructively with the London patient, in whom only CCR5-tropic provirus (and no CXCR4-tropic virus) was identified in CD4+ T-cell HIV-1 DNA before transplantation. The post-transplant CD4+ T cells did not express CCR5 and were susceptible only to X4 virus ex vivo, yet durable remission was achieved [10]. Taken together, these findings suggest that absence of an archived X4 reservoir may be necessary for CCR5∆32-based remission. Therefore, deep-sequencing analysis of viral tropism before transplantation may be useful when selecting suitable candidates, although this is not yet a formal requirement.

## 8. Implications for Pharmacologic CCR5 Modulation

The tropism limitation applies equally to reversible pharmacologic strategies. Maraviroc is the only licensed CCR5 antagonist and is active only against R5 virus. The clinical guidelines require documented CCR5 tropism before use. A co-receptor tropism assay should be performed whenever a CCR5 antagonist is considered and once X4- or D/M-tropic virus has ever been detected, CCR5 antagonists are considered permanently inactive and should not be used [32,33]. Use of a CCR5 antagonist in the presence of undetected X4/D-M virus increases the risk of virologic failure and resistance to companion agents [32,34]. Early tropism assays were less sensitive and could miss low levels of X4 variants. As a result, some patients with undetected X4 variants experienced rapid virologic failure after CCR5 antagonist treatment. Current assays can reliably detect CXCR4-using variants when they make up about 0.3% or more of the viral population [24,35]. By extension, JAK/STAT-mediated downregulation of CCR5 would exert no antiviral selective advantage against CXCR4-using virus and would be subject to the same tropism-dependent escape.

## 9. CCR5Δ32 and Graft-Versus-Host Disease

Recipients carrying the CCR5Δ32 allele develop acute GVHD less frequently than those with wild-type CCR5 (OR = 0.391, *p* = 0.023), an effect that is independent of other known risk factors including recipient age, donor type and conditioning regimen [36]. This protective effect is particularly pronounced when both recipient and donor carry the CCR5Δ32 allele with no acute GVHD observed in such pairs (0/11 vs. 70/151, *p* = 0.002) [36]. However, conflicting data exist; some animal models suggest that CCR5 absence on donor cells may impair regulatory T-cell function and paradoxically worsen GVHD in lethally irradiated settings [37].

## 10. CCR5 Gene Regulation by JAK/STAT Inhibitors

**Figure 3:**
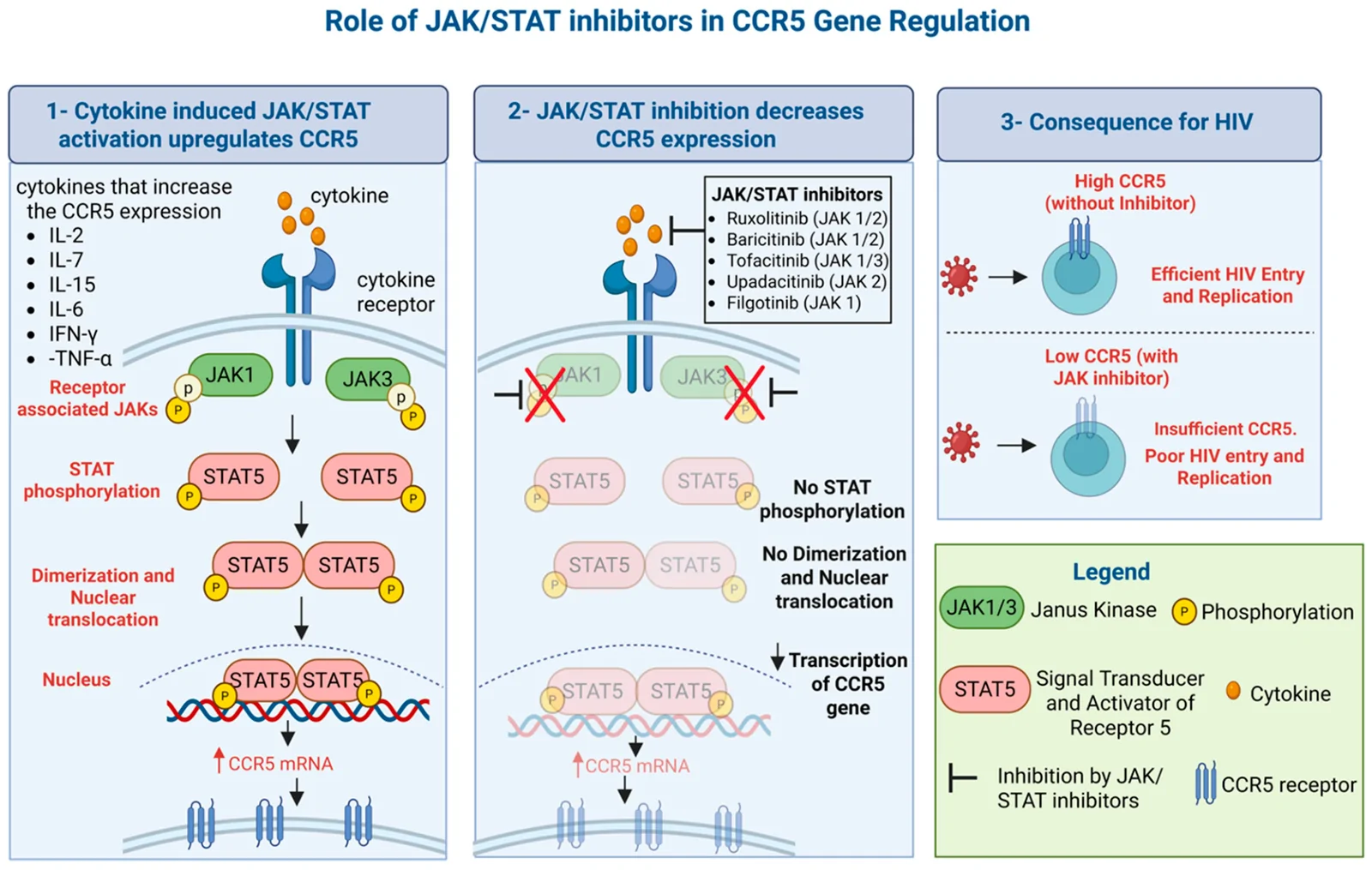
Regulation of CCR5 expression through the JAK/STAT signaling pathway.

The Janus kinase/signal transducer and activator of transcription (JAK/STAT) pathway is a principal intracellular signaling cascade activated by type I and type II cytokine receptors. Four JAK family members, i.e., JAK1, JAK2, JAK3 and TYK2, selectively associate with cytoplasmic domains of cytokine receptors and upon ligand binding, phosphorylate STAT proteins (STAT1–STAT6) which dimerize and translocate to the nucleus and regulate gene expression [38]. The CCR5 gene encoding the C-C chemokine receptor type 5 is transcribed from two promoters (Pu and Pd), with the proximal promoter Pd being used two- to five-fold more frequently in primary activated T cells [39]. Functional analysis of the minimal CCR5 promoter (nt −189 to +36) has identified two consensus STAT-binding sites, alongside binding motifs for NF-κB, AP-1, NF-AT and CD28RE. Mutagenesis of these STAT sites significantly impairs promoter activity, establishing a direct transcriptional link between STAT proteins and CCR5 expression [39] (Figure 3).

## 11. Bidirectional Relationship: CCR5 Ligand Signaling Through JAK/STAT

A landmark study systematically evaluated nine JAK/STAT inhibitors for their effects on CCR5 and CCR2 expression in human primary CD4+ T cells [2]. Six of nine inhibitors significantly reduced CCR5/CCR2 expression, with tofacitinib and ruxolitinib demonstrating inhibition of CCR5 expression even in CD4+ T cells from HIV-positive viremic patients [2]. Critically, CRISPR-mediated knockout experiments revealed that single knockouts of JAK2, STAT3, STAT5A and STAT5B each significantly reduced CCR2/CCR5 expression at both RNA and protein levels and various combinations of JAK/STAT knockouts produced additive reductions [2].

The downstream mediator serum and glucocorticoid regulated kinase 1(SGK1) has emerged as an additional regulatory node. SGK1 knockout affected CCR2/CCR5 gene expression, suggesting its involvement in the JAK/STAT-CCR5 regulatory axis [2]. SGK1 is itself a prominent transcriptional target of cytokine-induced JAK/STAT signaling with pharmacological JAK/STAT pathway inhibition abolishing STAT3 phosphorylation and SGK1 induction [40]. These findings position SGK1 as a signal amplifier within the JAK/STAT-CCR5 regulatory circuit [41].

## 12. Therapeutic Implications: HIV Cure and Beyond

The ability of JAK inhibitors to downregulate CCR5 has significant therapeutic implications. Ruxolitinib and tofacitinib demonstrate sub-micromolar inhibition of HIV-1 replication across lymphocytes and macrophages, with their combination increasing efficacy 53- to 161-fold over monotherapy [15]. Apart from direct antiviral effects, JAK inhibitors also block HIV reservoir seeding by reducing the frequency of CD4+ T cells harboring integrated HIV DNA, and phosphorylated STAT5 (pSTAT5) strongly correlates with increased levels of integrated viral DNA in vivo [16]. In the ACTG A5336 randomized, controlled trial, ruxolitinib (10 mg twice daily for 5 weeks) in virologically suppressed people with HIV was well tolerated and significantly decreased markers of immune activation (HLA-DR/CD38 on CD4+ T cells) and the anti-apoptotic protein BCL-2, which promotes reservoir cell survival [42].

A particularly instructive case linking JAK/STAT inhibition directly to transplantation outcomes is a 53-year-old man with myeloid sarcoma. He underwent allo-HSCT in 2018 from an HLA-matched donor carrying fully wild-type CCR5 (no CCR5Δ32 allele at all). Antiretroviral therapy was withdrawn 40 months post-transplant. The patient has since maintained undetectable plasma HIV-1 RNA for more than two years despite the complete absence of CCR5Δ32-mediated protection. Notably, the patient remained on continuous ruxolitinib after transplantation for management of chronic graft-versus-host disease. The authors propose that sustained JAK/STAT inhibition may have favored ongoing decay of the residual viral reservoir and limited viral reactivation hence offering the first direct clinical suggestion that JAK inhibitor therapy and allo-HSCT may act synergistically to sustain HIV remission even without CCR5 disruption [43].

CCR5 is implicated in pathologies beyond HIV-1 infection, including cancer metastasis where its mis- or altered expression in epithelial cells augments invasion, stemness and resistance to DNA-damaging agents and graft-versus-host disease (GVHD), where CCR5-mediated lymphocyte trafficking drives alloreactive tissue destruction [44,45]. The CCR5Δ32 mutation conferring near-complete HIV-1 resistance in homozygous carriers has been central to at least five documented HIV-1 cures through stem cell transplantation, though recent evidence demonstrates that CCR5Δ32-mediated resistance is not absolutely essential for durable remission, as a patient achieved sustained HIV-1 remission after transplantation from a heterozygous CCR5 wild-type/Δ32 donor [6,13]. These findings, combined with the pharmacological accessibility of the JAK/STAT pathway, makes the JAK inhibitors a promising approach for reversibly downregulating CCR5 expression without the ethical and safety concerns of permanent, irreversible gene editing [3,46].

## 13. Limitations and Future Directions

Despite these promising findings, durable HIV remission through JAK/STAT-directed CCR5 modulation faces two principal limitations. First, because JAK inhibitors act reversibly, sustained transcriptional suppression of CCR5 requires ongoing drug administration; discontinuation risks rebound of CCR5 expression and potentially, reservoir reactivation before the latent pool has been sufficiently depleted. Second, prolonged JAK/STAT inhibition carries a systemic immunosuppression burden including risk of viral reactivation (e.g., cytomegalovirus, Epstein–Barr virus, hepatitis B virus) and secondary opportunistic infection which must be weighed against therapeutic benefit with long-term use.

Overcoming these limitations will likely require combination strategies that pair JAK/STAT-mediated microenvironment modulation with approaches that confer more durable or permanent protection. These include CRISPR/Cas9-mediated disruption of CCR5 in autologous or donor hematopoietic stem cells to establish permanent co-receptor resistance without the morbidity of allogeneic transplantation; strategies that augment natural killer (NK) cell-mediated antibody-dependent cellular cytotoxicity (ADCC) to clear persistently or reactivated infected cells, an approach supported by the potent ADCC activity observed in the heterozygous CCR5Δ32 transplant recipient described above [14], and immunotherapy innovations integrating broadly neutralizing antibodies (bNAbs) with CAR-T cell approaches to achieve multi-layered, durable reservoir control without lifelong immunosuppression. Together, these combinatorial strategies may extend the reversible, pharmacologically accessible advantages of JAK/STAT inhibition toward a scalable and durable HIV cure.

## Figures and Tables

**Table 1:** Cure and remission cases achieved through allogeneic HSCT.

Patient	Underlying Malignancy	Donor CCR5 Genotype	Conditioning/ Transplant Approach	Donor Chimerism	ART Interruption (ATI)	HIV Remission/Outcome
Berlin patient	AML	CCR5Δ32/Δ32	Two allo-HSCTs, myeloablative conditioning with total body irradiation	Not specified	Yes	Undetectable viral load without ART; remission maintained until death from recurrent AML 13 years later
London patient	Hodgkin Lymphoma	CCR5Δ32/Δ32	Single allo-HSCT, reduced-intensity conditioning, no irradiation	Not specified	Not specified	Sustained HIV remission
Düsseldorf patient	AML	CCR5Δ32/Δ32	Allo-HSCT	Not specified	Yes	No replication-competent virus despite sporadic HIV-1 DNA; 4 years of sustained remission after ART
New York patient	AML	CCR5Δ32/Δ32 cord blood donor	Haplo-cord transplantation (CCR5Δ32/Δ32 cord blood+ haploidentical adult stem cells)	100% CCR5Δ32/Δ32 cord blood chimerism by week 14	Not specified	Sustained undetectable HIV-1 load without ART
City of Hope patient	Hematological malignancy	CCR5Δ32/Δ32	Reduced-intensity conditioning	Not specified	Not specified	HIV cure/remission achieved despite age 63 years
2026 sibling donor case	Myelodysplastic syndrome	CCR5Δ32/Δ32	Allo-HSCT from CCR5Δ32/Δ32 sibling donor	Full donor chimerism in gut tissue	Not specified	No replication-competent virus; 5 years of HIV remission
Heterozygous donor case	Hematological malignancy	CCR5 WT/Δ32	Allo-HSCT	Reconstituted immune system with functionally active CCR5	Not specified	>6 years of HIV-1 remission, demonstrating that complete CCR5 ablation is not essential

## Data Availability

No new data were created or analyzed in this study. Data sharing is not applicable to this article.
